# Urinary balantidiasis in a patient with systemic lupus erythematosus and lupus nephritis: a case report

**DOI:** 10.1186/s13256-020-02389-7

**Published:** 2020-05-28

**Authors:** Pongsakorn Martviset, Kridsada Sirisabhabhorn, Supaporn Pumpa, Pochong Rhongbutsri, Aree Taylor, Walter R. J. Taylor

**Affiliations:** 1 Division of Parasitology, Department of Preclinical Sciences, Faculty of Medicine, Thammasat University, Pathumthani, 12120 Thailand; 2 Department of Medical Technology Laboratory, Thammasat University Hospital, Pathumthani, Thailand; 3 Mahidol Oxford Tropical Medicine Research Unit, Bangkok, Thailand; 4 Division of Tropical and Humanitarian Medicine, University Hospitals of Geneva, Geneva, Switzerland

**Keywords:** Urinary balantidiasis, Systemic lupus erythematosus, Prednisolone

## Abstract

**Background:**

*Balantidium coli*, a parasitic unicellular ciliate, often causes asymptomatic balantidiasis of the colon, but extraintestinal disease may occur rarely in immunosuppressed individuals. Renal balantidiasis associated with systemic lupus erythematosus has not been reported before.

**Case presentation:**

We present a case of a 48-year-old Thai woman who presented with nephrotic syndrome due to systemic lupus erythematosus–related nephritis. Initially, few *B. coli* cysts were found in urine sediment, but these increased substantially following treatment with prednisolone. She made an uneventful recovery with 10 days of oral tetracycline therapy. No *B. coli* cysts were found in her stool.

**Conclusion:**

The route of infection in our patient was unclear but is likely to have been orofecal. Neither her infection nor its treatment caused a deterioration in her renal function.

## Background

*Balantidium coli* (*Neobalantidium coli*) is a large, unicellular, ciliated parasite that infects mainly the gastrointestinal tract of humans and several mammals, such as wild pigs, cattle, sheep, and goats [1]. *B. coli* has two stages: the trophozoite and cyst. Trophozoites are irregularly shaped and are the living stage of the parasite, whereas the cyst is the infective stage and has a thick wall for protection from the environment [2–4].

Humans can be infected by ingesting mature cysts that infect water and food. Once ingested, the cyst releases trophozoites in the duodenum, which then mature and migrate to the colon, where they replicate by transverse binary fission and, less frequently, by conjugation. Some trophozoites may invade the colonic wall and multiply to cause colonic ulcers and a dysenteric syndrome similar to that of *Entamoeba histolytica*. Trophozoites generally encyst in the colon and are passed into the environment with the feces [2, 4].

The estimated global prevalence rates in humans vary between 0.02% and 1%, with higher rates reported in animals, such as pigs, in which is as high as 93% [2, 4]. Regions and countries with the highest human prevalence rates are Latin America, the Philippines, Papua New Guinea, and the Middle East, where rates of up to 29% have been reported in pig farmers [2, 5–7]. The majority of infections are asymptomatic and are probably due to avirulent or low-virulence strains. Dysentery is uncommon and is thought to be related to the immune status of the individual [8–10]. Extraintestinal balantidiasis is rare but has been reported in several organs, such as the liver, lungs, and genitourinary tract, in immunodeficient and otherwise healthy patients [8, 11, 12]. There are few case reports of urinary balantidiasis occurring in patients with steroid-treatedchronic obstructive pulmonary disease, patients with chronic renal failure, and in two cases without underlying diseases [13]. There is no previous report of urinary balantidiasis in patients with systemic lupus erythematosus (SLE). We report a case of a patient with SLE who was found to have incidental urinary balantidiasis on the basis of routine urine testing.

## Case presentation

A 48-year-old Thai woman with congenital mutism presented with a 3-month history of intermittent swelling of both lower limbs. She was otherwise well and did not report joint pain, headache, photophobia, rash, dyspnea, orthopnea, palpitations, hair loss, or bowel symptoms. Her appetite was normal. She had no history of clinically significant underlying diseases; she was not receiving any drug treatments; and she did not drink alcohol or smoke. Her physical examination revealed that she was well; her weight was 41 kg, and her body temperature was 37.2 °C, pulse rate was 103 beats/minute, blood pressure was 116/70 mmHg, and respiratory rate was 20 breaths/minute. She had no abnormal findings of her head, ears, eyes, nose, throat, heart, lungs, and abdomen. The only abnormal sign was bilateral pitting edema 3+ below the knee without erythema and increased warmth.

Routine laboratory tests (Table 1) showed that she had a mild microcytic anemia (hemoglobin 9.4 mg/dl, mean corpuscular volume 73 fl). Her serum total protein and albumin concentrations were low, but she had hyperglobulinemia and raised liver enzymes (aspartate aminotransferase, alanine aminotransferase, and alkaline phosphatase). Her serum creatinine was 0.82 mg/dl, for an estimated glomerular filtration rate (eGFR) of 85.49 ml/min/1.73 m^2^, blood urea nitrogen (BUN) 21.20 mg/dl, sodium 137 mEq/L, potassium 4.0 mEq/L, and chloride 103 mEq/L. Her hepatitis (anti-hepatitis C virus antibodies, hepatitis B surface antigen) and human immunodeficiency virus serology results were negative.

**Table 1:** Selected routine laboratory findings

Parameters	Visit 1	Visit 2	Visit 3	Visit 4	Post-treatment	Reference values
Hematology						
Hb (g/dl)	9.4	9.2	8.7	–	–	12–15
Hct (%)	29.7	28.4	26.3	–	–	36–45
MCV (fl)	73	68	65	–	–	80–96
WBC count (× 10^9^ cells/L)	6.8	6.4	5.3	–	–	4–10
Biochemistry						
Total protein (mg/dl)	6.2	–	–	–	–	6.4–8.3
Albumin (mg/dl)	1	–	–	–	–	3.5–5
Globulin (mg/dl)	5.2	–	–	–	–	2.3–2.8
AST (IU/L)	159	–	–	–	–	< 42
ALT (IU/L)	72	–	–	–	–	< 40
ALP (IU/L)	258	–	–	–	–	32–92
Creatinine (mg/dl)	–	0.82	0.84	1.48	0.88	0.6–1.3
eGFR (ml/min/1.73 m^2^)	–	85.49	83.03	41	84.55	> 125
Blood urea nitrogen (mg/dl)	–	21.2	23.27	37.5	–	10–20
Urinalysis						
Color	Yellow	Yellow	Yellow	Yellow	Yellow	Colorless to amber
Appearance	Turbid 2+	Turbid 2+	Turbid +	Turbid +	Clear	Clear
Specific gravity	1.021	1.04	1.022	1.017	1.01	1.003–1.030
pH	6	6	6	7.5	6	5.5–6.5
Protein	3+	4+	4+	4+	3+	Negative
Blood	3+	3+	2+	2+	1+	Negative
Leukocyte esterase	1+	2+	2+	2+	Negative	Negative
RBC (cells/HPF)	20–30	20–30	5–10	30–50	5–10	0–1
WBC (cells/HPF)	5–10	> 100	> 100	> 100	5–10	0–5
Fine granular cast (/LPF)	0–1	–	–	–	–	Negative
Coarse granular cast (/LPF)	5–10	–	–	–	–	Negative
*B. coli* trophozoites	Negative	+	+	3+	Negative	Negative
Spot urine						
Total protein (mg/dl)	1339.4	–	–	1679	–	2–8
Creatinine (mg/dl)	143.79	–	491.1	100.34	–	25–400
24 h urine protein (g)	9.3	–	4.9	–	–	< 0.15
Stool examination	–	–	Negative	Negative	Negative	Negative

*Abbreviations:ALP* Alkaline phosphatase, *ALT* Alanine aminotransferase, *AST* Aspartate aminotransferase, *eGFR* Estimated glomerular filtration rate, *Hb* Hemoglobin, *Hct* Hematocrit, *HPF* High-power field,*LPF* Low-power field, *MCV* Mean corpuscular volume, *RBC* Red blood cells, *WBC* White blood cells

Her urine was yellow and turbid, and dipstick urinalysis (Roche Diagnostics, Mannheim, Germany) demonstrated a specific gravity of 1.021, pH 6.0, protein 3+, blood 3+, red blood cells 20–30 cells/high-power field (HPF), white blood cells 5–10 cells/HPF. Urine microcopy of the urine sediment showed few fine granular casts 0–1/low-power field (LPF) and coarse granular casts 5–10/LPF. By spot urine, her total protein and creatinine were 1.339.4 mg/dl and 143.79 mg/dl, respectively.

Suspecting chronic kidney disease secondary to an autoimmune disease, we performed additional investigations, which revealed a positive antinuclear antibody. For the titer of 1280, it revealed the homogeneous and fine speckled patterns meanwhile the nucleolar, peripheral, and cytoplasm patterns were observed in the titer of less than 80. The patient’s C3 complement concentration was 50.0 mg/dl (normal 81–157 mg/dl), and her C4 complement concentration was < 8.0 mg/dl (normal 13–39 mg/dl). Her urinary protein excretion over 24 hours was 9.3 g, meeting the case definition of nephrotic proteinuria. The result of a second urine analysis was similar to that of the first analysis (Table 1). The patient was diagnosed with clinically suspected SLE with lupus nephritis. A referral for a renal biopsy was made, and she was prescribed prednisolone 1 mg/kg. Two days later, a third urinalysis was performed (Table 1). In addition to similar results as before, several rapidly moving, large, ovoid-shaped ciliated parasites were seen by video clip/light microscopy; these findings were confirmed by two additional microscopic analyses of her urine sediment. No antiparasitic treatment was given, and she was asked to come for follow-up 2 weeks later.

**Fig. 1:**
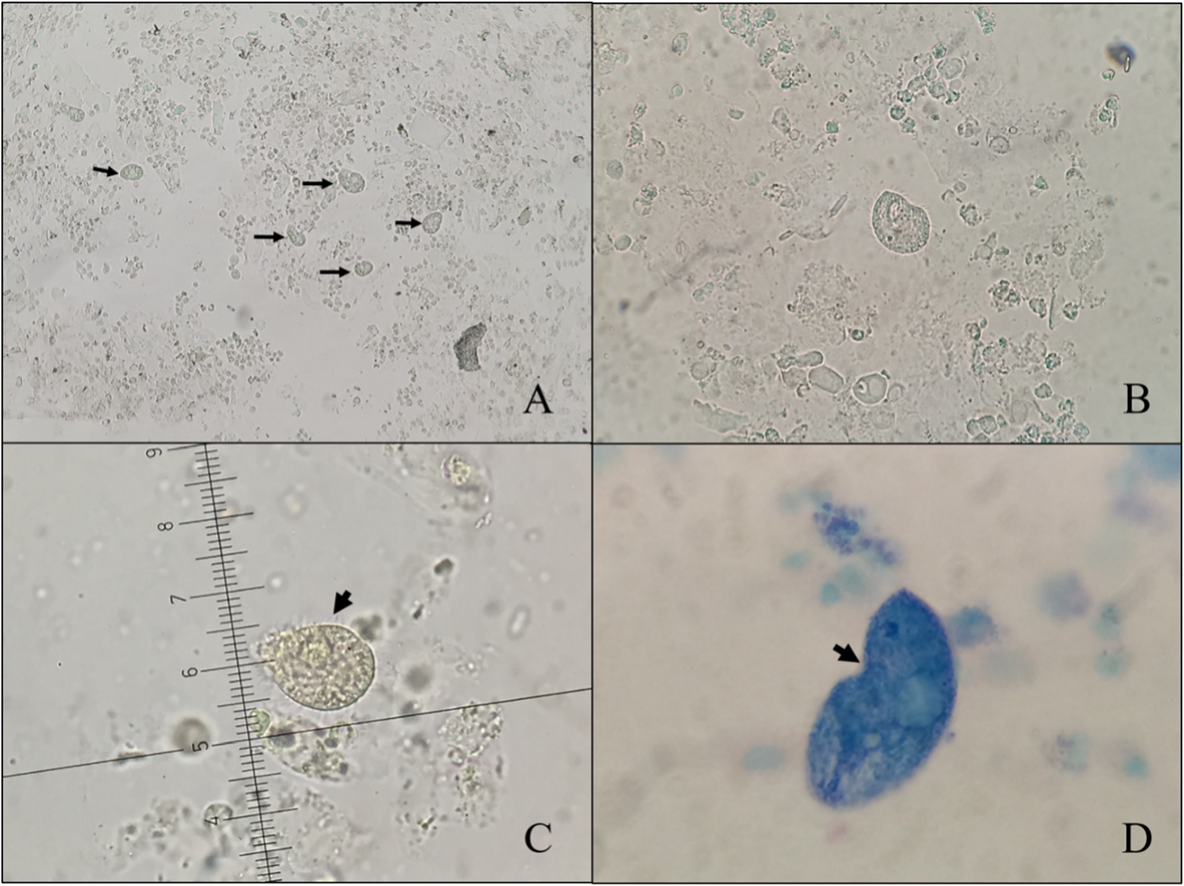
Detection of *B. coli* trophozoites in urine samples of our patient with systemic lupus erythematosus. **a** Several trophozoites (*arrows*) of different sizes in one low-power field (× 100 magnification). **b** A single trophozoite and **c** ciliated (*arrow*) trophozoite with a micrometer scale, measuring 30 × 37.5 μm (× 400 magnification). **d** Wright-Giemsa staining of trophozoite showing cytostome (*arrow*) and several contractile vacuoles

At follow-up, a fourth urine analysis was done (Table 1), and a spot urine protein (1679.0 mg/dl) and creatinine (100.34 mg/dl) were measured. Serum BUN and creatinine were 37.5 and 1.48 mg/dl, respectively. Numerous motile, ciliated trophozoites were seen in three consecutive urine samples by wet preparation and Wright-Giemsa staining that were identified as *B. coli* (Fig. 1). Three daily stool examinations by formalin-ethyl acetate concentration were negative for ova, cysts, and parasites. She was prescribed tetracycline 500 mg four times daily for 10 days, after which the result of a post-treatment urine examination was negative for *B. coli*.

## Discussion

Balantidiasis is usually an asymptomatic infestation of the colon, but symptoms may manifest in patients who are immunosuppressed, and such patients may also experience systemic involvement [2, 4]. We hypothesize that our patient with SLE with renal *B. coli* was an asymptomatic carrier, that invasion of the urinary tract probably took place because of her SLE, and that this invasion increased after prednisolone was prescribed, as evidenced by a greater number of *B. coli* trophozoites in her urine analysis. She made an uneventful recovery with 10 days of tetracycline.

Urinary balantidiasis is rare. The first reported case in 2007 was in a 60-year-old Indian man who complained of fever, lower abdominal pain, dysuria, and urinary frequency [14]. He had no underlying disease and no history of contact with pigs [15]. In other case reports, all patients had an underlying immunocompromising disease such as diabetes mellitus, steroid-treated chronic obstructive pulmonary disease, and chronic kidney disease [14–18], where the urinary balantidiasis was accidentally found during the urinalysis.

Our patient lived in urban Bangkok and had no contact with pigs. Thus, the most likely route of infection was ingesting infective cysts via contaminated water or fresh vegetables or by eating undercooked (that is, improperly grilled) pig intestine. Grilled pig intestine is a very popular dish in some parts of Thailand. Her infection responded well to tetracycline, the recommended first-line treatment. Some authorities recommend that tetracycline should not be used in patients with impaired renal function, because it may cause an increased concentration of BUN due to an antianabolic and diuretic effect [19, 20]. Moreover, impaired excretion results in increased tetracycline accumulation and potential toxicity [21]. Our patient’s eGFR was only mildly depressed, she was receiving treatment with steroids, and subsequent serum creatinine measurements did not indicate a deterioration in her renal function due to tetracycline. Alternatives to tetracycline include metronidazole and iodoquinol.

The diagnosis of *B. coli* in our patient was made on the basis of clearly identified morphological features and motility using direct wet preparation, staining, and video microscopy. Other common urine parasites, such as *Trichomonas vaginalis* (the most common parasite in the genitourinary tract) and *E. histolytica*, are readily distinguished on the basis of morphology. *B. coli* is the only ciliated parasite without flagellae, and its trophozoite movement is unique, a rapid spiral movement produced by the cilia. *T. vaginalis* trophozoite is the only pathogenic flagellate of the urinary tract; it has a characteristic long, filamentous flagellum and displays jerky movements. *E. histolytica* trophozoites are smaller (8–30 μm). Those of *B. coli* do not have a macronucleus or cilia, and their mobility is characterized by use of their ectoplasmic pseudopods; their cysts are also smaller (~10–20 μm) than *B. coli* cysts, whereas *T. vaginalis* does not have a cyst stage.

## Conclusion

Urinary balantidiasis is rare, is often associated with immunosuppression, and is commonly an incidental finding. It does not usually cause serious renal complications, as was the case in our patient with SLE nephritis. A video recording and Wright-Giemsa staining were useful for the diagnosis. Prevention of *B. coli* infection lies with better sanitation, providing clean water, avoiding the consumption of undercooked pig intestine, and good hand hygiene, especially for those who work with pigs.


**Additional file 1.***B. coli* trophozoite rotary-movement in 400X magnification.



**Additional file 2.** Direct wet smear of patient urine specimen in 0.85% NaCl showing several live *B. coli* trophozoites in 100X magnification.


## Data Availability

All the data regarding the findings are available within this report.
